# Research on the Coupling Effect of NBTI and TID for FDSOI pMOSFETs

**DOI:** 10.3390/mi15060702

**Published:** 2024-05-25

**Authors:** Hao Wei, Hongxia Liu, Shulong Wang, Shupeng Chen, Chenyv Yin, Yaolin Chen, Tianzhi Gao

**Affiliations:** Key Laboratory for Wide-Band Gap Semiconductor Materials and Devices of Education, School of Microelectronics, Xidian University, Xi’an 710071, China; weihnb666@163.com (H.W.); slwang@xidian.edu.cn (S.W.); spchen@xidian.edu.cn (S.C.); yin_chenyu@163.com (C.Y.); cylin@stu.xidian.edu.cn (Y.C.); gaotianzhisoso@163.com (T.G.)

**Keywords:** NBTI, TID, coupling effect of NBTI and TID, FDSOI, simulation

## Abstract

The coupling effect of negative bias temperature instability (NBTI) and total ionizing dose (TID) was investigated by simulation based on the fully depleted silicon on insulator (FDSOI) PMOS. After simulating the situation of irradiation after NBT stress, it was found that the NBTI effect weakens the threshold degradation of FDSOI PMOS under irradiation. Afterward, NBT stress was decomposed into high gate voltage stress and high-temperature stress, which was applied to the device simultaneously with irradiation. The devices under high gate voltage exhibited more severe threshold voltage degradation after irradiation compared to those under low gate voltage. Devices at high temperatures also exhibit more severe threshold degradation after irradiation compared to devices under low temperatures. Finally, the simultaneous effect of high gate voltage, high temperature, and irradiation on the device was investigated, which fully demonstrated the impact of the NBT stress on the TID effect, resulting in far more severe threshold voltage degradation.

## 1. Introduction

With the development of Moore’s Law and the application of integrated circuits in the aerospace field, the non-ideal effects of devices in radiation environments are receiving increasing attention. The stability of bulk silicon technology is poor in radiation environments, and as the process size is scaled down, the short-channel effect of bulk silicon devices becomes increasingly significant [1]. The fully depleted silicon on insulator (FDSOI) technology, due to the presence of its buried oxygen (BOX) layer, has advantages in resisting single particle effect and transient dose rates, and to some extent improves the short-channel effect [2,3,4,5]. However, as the process size continues to be scaled down, the gate dielectric layer of the MOS device becomes thinner and thinner, and the FDSOI device still faces reliability issues that exist in bulk silicon devices, including the NBTI effect [6,7]: when the MOS device is under high temperature and high gate voltage stress, the Si-H bonds at the Si/SiO_2_ interface will be broken and form interface traps. For PMOS, the interface traps will capture holes and turn into positive interface trap charges, and the oxide trap charge is also generated during this process. In addition, although the gate dielectric layer has become thinner, the FDSOI device still suffers from the TID effect because of the BOX layer [8,9,10]. The physical processes of the TID effect are as follows: Radiation causes the peripheral electrons to break free from the atom and become free electrons. When radiation acts on the oxide of the semiconductor device, it will cause the generation of electron–hole pairs. Part of the radiation-induced electron–hole pairs will recombine, and as for the rest of the electron–hole pairs, the electrons drift out of the oxide layer in a very short time under the applied electric field due to their higher mobility, while the holes migrate slowly to the Si/SiO_2_ interface. During this process, part of the holes will be captured by traps, and thus, positive oxide trap charges and positive interface trap charges are formed. As the radiation dose continues to increase, the trap charge accumulates, and the degradation of the device becomes more severe; this phenomenon is known as the TID effect. It is worth noting that both the NBTI effect and the TID effect lead to the generation of oxide trap charge and interface trap charge, and the degradation of the device’s electrical parameter caused by the NBTI effect and the TID effect are very similar.

The mechanisms of the TID effect and the NBTI effect for SOI devices have been systematically studied [11,12,13]. However, the research on the degradation of SOI devices under the combined effect of TID and NBTI is relatively poor. The work of Wang et al. studied the threshold voltage degradation of a bulk silicon device under the combined effect of NBTI and TID [14] and found that the time exponent of the threshold voltage degradation curve of the irradiated device under NBT stress significantly increased compared to the un-irradiated device, which means irradiation before NBT stress will accelerate the threshold voltage degradation of the device during NBT stress. Based on the PDSOI device, Peng et al. investigated the impact of BOX Si+ implantation TID hardening technology on the NBTI effect [15] and their work showed that more damage-related traps exist near the gate oxide/silicon interface for the radiation-hardened device, which accelerates the threshold voltage shift during NBT stress. The impact of the TID effect on the NBTI effect of the PDSOI device was investigated in a further paper by Peng et al. [16], and it was found that the irradiated device shows a smaller threshold voltage shift than the un-irradiated device under the same NBTI stress at the early stress stage, but the irradiation also increases the time exponent and leads to a significant reduction in the lifetime of the device. The influence of TID irradiation on the NBTI trap characteristics was also analyzed in this paper. Liu et al. investigated not only the impact of the TID effect on the NBTI effect but also the impact of the NBTI effect on the TID effect [17], and in addition to the degradation of the threshold voltage, the degradation of the transconductance and the subthreshold swing were also discussed. In the above works on the coupling effect of NBTI and TID, the method of studying the coupling effect of NBTI and TID is generally to apply NBT stress first and then irradiation, or to apply NBT stress after irradiation to study the degradation of the device after these two successive stresses. However, semiconductor devices used in space face a very harsh and complex operating environment, and in many cases, they are subjected to thermal stress, electrical stress, and radiation at the same time; accordingly, it is necessary to study the degradation of the device under the simultaneous stress of high temperature, high gate voltage, and radiation.

In this paper, the coupling effect of NBTI and TID on FDSOI PMOS is investigated by a simulation. In order to keep the research close to the actual working environment of the device, in addition to studying the influence of the NBTI effect on the TID effect by simulating the situation of irradiation after NBT stress, the situation of simultaneous NBT stress and irradiation on the FDSOI PMOS was also investigated. The threshold voltage shift of FDSOI PMOS under the above stress conditions was extracted and analyzed. The trap charge generation of FDSOI PMOS during stress was also discussed to further understand the coupling effect of NBTI and TID on FDSOI PMOS. For the FDSOI process, the thickness of the gate oxide layer was reduced to around 2 nm, and such a thin gate oxide layer weakens the impact of the gate oxide trap charge on the device, so the discussion of the trap charge in this paper focuses on the front-gate interface trap charge and oxide trap charge in the BOX layer.

## 2. Simulation Details

### 2.1. Device Model and Electrical Parameter Extraction

**Figure 1:**
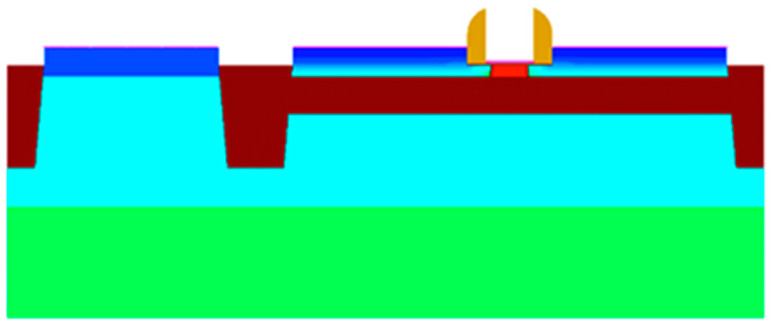
Device model of FDSOI PMOS.

The Simulation software used in this work is Sentaurus TCAD (Version: 2018.06). Figure 1 shows the device model of FDSOI PMOS, with a 7 nm top silicon film and a 25 nm BOX layer. The thickness of the gate oxide is 2 nm, and the doping information is listed in Table 1.

**Figure 2:**
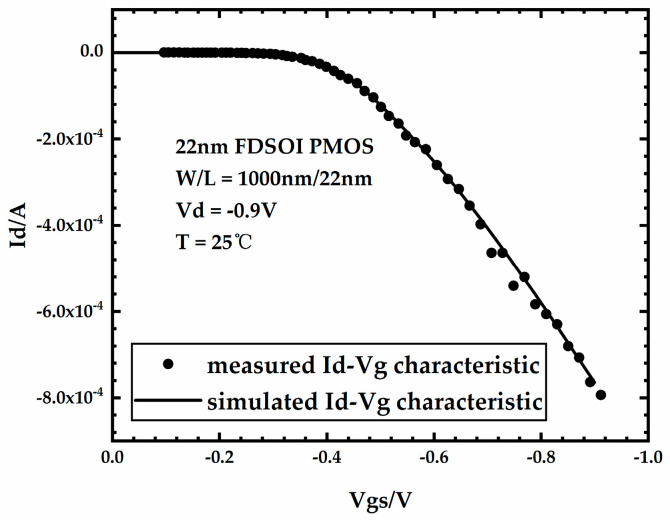
Id−Vg characteristic of FDSOI PMOS.

The bias state of the device during the extraction of I-V characteristics is Vd = −0.9 V and Vs = Vb = 0 V, and Vg was swept from 0 V to −0.9 V. The threshold voltage was extracted using the constant current method where the drain current reached (W/L) × 10^−7^ A. Figure 2 shows the simulated and measured [18] Id-Vg characteristics of the FDSOI PMOS device. The error between the simulated Id-Vg curve and the measured Id-Vg curve is very small, which demonstrates the accuracy of the device model.

### 2.2. Physical Model for Simulating NBTI Effect

The Trap Degradation Model (TDM) built in Sentaurus Tcad was used to simulate the NBTI effect. TDM is a simulation model for the NBTI effect developed based on the reaction-diffusion theory [19], which expresses the diffusion of H in the oxide layer through the following equations:

(1) $$D\frac{dN_{H}}{dx}=\frac{{dN}_{hb}}{dt}\;\;\;\;\;\;\;\;\;x=0$$

(2) $$\frac{dN_{H}}{dt}=D\frac{d^{2}N_{H}}{dx^{2}}\;\;\;\;\;\;\;0<x<xp$$

(3) $$D\frac{dN_{H}}{dx}=-k_{p}\left(N_{H}-N_{H}^{0}\right)\;\;\;\;\;\;\;x=x_{p}$$

where $N_{H}$ is the concentration of hydrogen in the oxide layer, $N_{hb}$ is the concentration of unbroken Si-H bonds, D is the diffusion coefficient of hydrogen in the oxide layer, which can be expanded as $D=D_{0}exp(-\varepsilon_{H}/kT)$, characterizing the effect of temperature and activation energy on the diffusion of hydrogen in the oxide layer, $N_{H}^{0}$ is the initial concentration of hydrogen in the oxide layer, x=0 represents the position of the interface between the channel and the oxide layer, and x=xp represents the position of the interface on the other side of the oxide layer (the value of xp should be set equal to the thickness of the oxide layer). $k_{p}$ is the surface recombination velocity at $x=x_{p}$.

(4) $$\begin{array}{c} \frac{dN_{hb}}{dt}=-vN_{hb}+\gamma (N-N_{hb})\\ \gamma =\gamma_{0}\frac{N_{H}}{N_{H}^{0}}\;,\;\;\;\gamma_{0}=v_{0}\frac{N_{hb}^{0}}{N-N_{hb}^{0}} \end{array}$$

The interface trap density $N_{it}$ meets Equation (4), where N is the sum of the densities of all unbroken and broken Si-H bonds at the interface ($N_{it}=N-N_{hb}$), $N_{hb}^{0}$ is the density of the initial unbroken Si-H bonds, v is the de-passivation rate (the rate of Si-H bond breakage), γ is the passivation rate (the Si-bonds and hydrogen re-form into Si-H bonds), and $v_{0}$ and $\gamma_{0}$ are de-passivation and passivation constants, respectively. The physical meaning expressed by Equation (4) is straightforward, i.e., the rate of variation of the Si-H bond concentration is equal to the Si-H bonds regenerated due to passivation minus the Si-H bonds broken due to de-passivation.

It can be noted that the influence of stress conditions such as temperature and the electric field on the NBTI effect is not included directly in Equations (1)–(4). This is because it is contained in the de-passivation rate v, which can be expanded as Equation (5):

(5) $$\begin{array}{c} v=v_{0}exp(\frac{\varepsilon_{A}^{0}}{{kT}_{0}}-\frac{\varepsilon_{A}^{0}+∆\varepsilon_{A}}{\varepsilon_{T}})\\ ∆\varepsilon_{A}={-\partial }_{⊥}|F_{⊥}{|}^{\rho }+\beta \varepsilon_{T}\frac{N-N_{hb}}{N-N_{hb}^{0}}\\ \varepsilon_{T}=kT,\;\;\;\;\beta =\beta_{0}+\beta_{⊥}\;F_{⊥} \end{array}$$

where $\varepsilon_{A}^{0}$ is the activation energy of the Si-H bond breaking without stress, $∆\varepsilon_{A}$ is the variation in the activation energy of the Si-H bond breaking due to the NBT stress, $F_{⊥}$ is the electric field perpendicular to the interface between the channel and the oxide layer, $\partial_{⊥}$, $\beta_{⊥}$, $\beta_{0}$, and ρ are the electric field enhancement parameters, k is the Boltzmann constant, and T represents the temperature in Kelvin.

**Figure 3:**
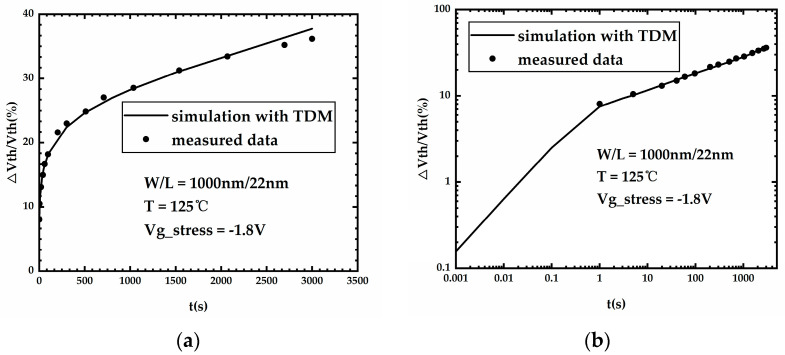
Threshold voltage shift of the FDSOI PMOS versus NBT stress time obtained from measurements in the literature and simulation. (**a**) Linear coordinate. (**b**) Double-logarithmic coordinate.

The parameters of TDM were carefully calibrated to ensure the accuracy of the simulation, and Table 2 shows the parameter value of TDM after calibration. Figure 3 is a plot of the threshold voltage shift of the FDSOI PMOS versus NBT stress time obtained from measurements in the literature [18] and simulation with the calibrated TDM, which shows that the TDM can aptly simulate the NBTI effect, and the error between the threshold voltage shift obtained from the simulation and the actual measurement does not exceed 10%.

### 2.3. Physical Model for Simulating TID Effect

The TID effect was simulated by the Gamma Radiation Model (GRM), which is also a built-in model in Sentaurus TCAD. In GRM, the generation of electron–hole pairs due to radiation is an electric field–dependent process [20] and is modeled as follows:

(6) $$G_{r}=g_{0}\times D\times Y(F)$$

where $G_{r}$ is the generation rate of electron–hole pairs during irradiation, $g_{0}$ is the electron–hole pair generation constant, D is the dose rate, and Y(F) is used to model the influence of the electric field on the generation rate of electron–hole pairs during irradiation, which can be expanded as Equation (7):

(7) $$Y(F)={\left(\frac{F+E_{0}}{F+E_{1}}\right)}^{m}$$

where $E_{0}$, $E_{1}$, and m are constant parameters.

In addition to GRM, other settings are required to simulate the TID effect. In Sentaurus TCAD, it is assumed that electron–hole pairs will not appear in oxide; however, the degradation due to the TID effect is caused by the trapping of holes in the oxide layer. This problem can be solved by replacing the material of the oxide layers in the device model from SiO_2_ to OxideAssemiconductor, which is a material defined by Sentaurus Tcad for such a situation. The capturing of holes by the oxide trap is also a physical process of TID degradation, while the capture rate of holes by the oxide trap is positively correlated with the capture cross-section. The capture cross-section can be expressed as follows [21]:

(8) $$\sigma_{n,p}=\sigma_{n,p}^{0}{\left(1+a_{1}{\left|F\right|}^{p_{1}}+a_{2}{\left|F\right|}^{p_{2}}\right)}^{p_{0}}$$

where $a_{1}$, $a_{2}$, $p_{0}$, $p_{1}$, and $p_{2}$ are constant parameters and F is the electric field in the oxide layer. From Equation (8), it can be found that the capture cross-section is mainly affected by the electric field.

**Figure 4:**
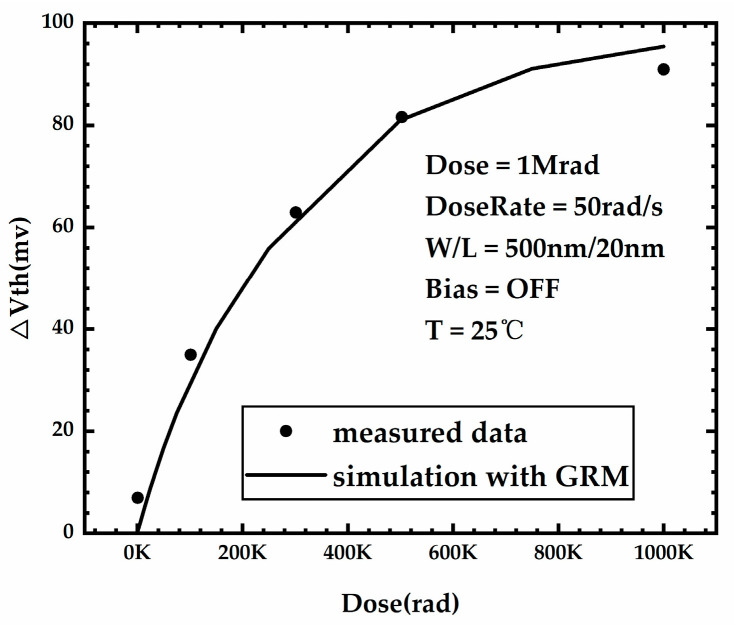
Threshold voltage shift of the FDSOI PMOS versus irradiation dose obtained from measurement in the literature and simulation.

To ensure the accuracy of the simulation, the parameters of the physical model for simulating the TID effect also need to be calibrated, and Table 3 shows the parameter value of the physical model for simulating the TID effect after calibration. Figure 4 is a plot of the threshold voltage shift of the FDSOI PMOS versus irradiation dose obtained from measurements in the literature [22] and simulation with the calibrated model. There is no more than 15% error between the simulation and the measurements.

## 3. Results and Discussion

In this section, the coupling effect of NBTI and TID was simulated based on the TDM and GRM. The threshold voltage shift of the devices was extracted from the simulation results, and the variation of trap charges of the devices was also extracted and analyzed.

### 3.1. NBTI after TID

**Figure 5:**
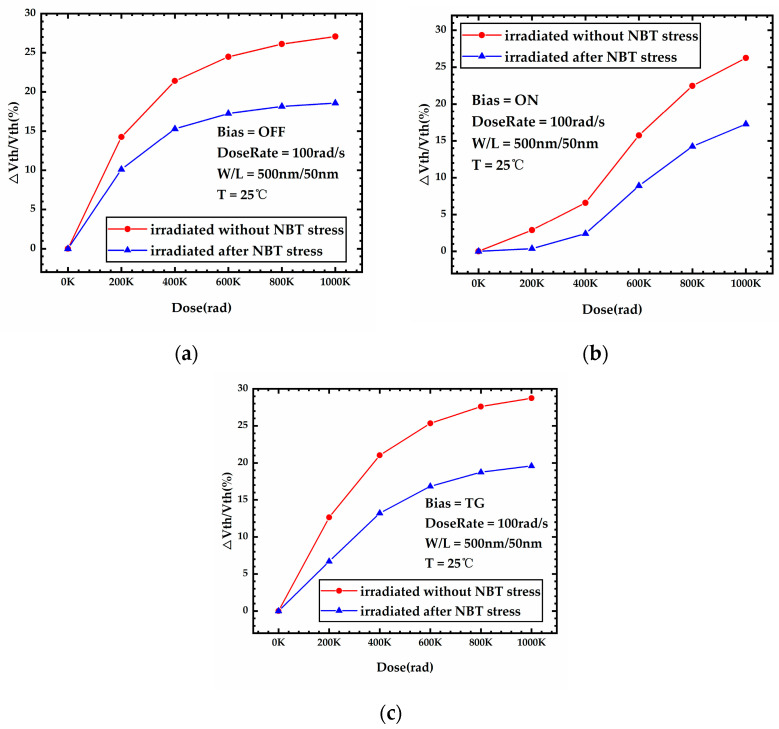
Threshold voltage shift percentage during irradiation for the devices without and after NBT stress; device is (**a**) OFF bias, (**b**) ON bias, and (**c**) TG bias during irradiation.

**Figure 6:**
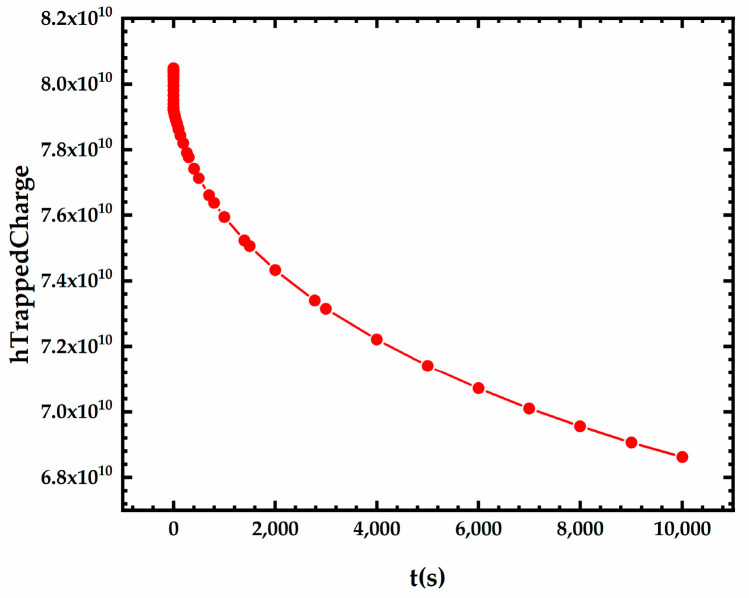
Variation of the front-gate interface trap charges during irradiation for device after NBT stress.

Figure 5 shows the variation curves of the threshold voltage shift percentage during irradiation for the devices without and after 3000 s NBT stress, and the bias conditions of the devices during irradiation are listed in Table 4. The temperature and gate voltage of the NBT stress are 125 °C and −1.8 V, respectively. Figure 5 shows that for the devices under all three bias conditions of OFF, ON, and TG, the threshold voltage shift of the devices after NBT stress is less severe than that of the devices without NBT stress at any irradiation dose from 0 to 1 Mrad. This is because the recovery of the NBTI effect occurs at the same time as the irradiation. Figure 6 shows the variation of the trap charge in the front-gate interface during irradiation for the device after 3000 s NBT stress. As shown in Figure 6, a large amount of interface trap charge was formed at the front-gate interface of the device after 3000 s NBT stress. While subsequent irradiation is carried out under a low temperature and low gate voltage, the Si-H bonds that have been broken during previous NBT stress will be re-passivated and the interface trap charge will slowly decrease with time. The threshold voltage recovery due to the reduced interface trap charge compensates for part of the threshold voltage shift during irradiation. Therefore, the threshold voltage shift of the device after NBT stress is less severe than that of the device without NBT stress.

### 3.2. Simultaneous Effect of NBTI and TID

NBT stress refers to high temperature and high gate voltage stress, so the simultaneous effect of NBT stress and TID is to bias the device to the ON state with high gate voltage and to irradiate the device under high-temperature conditions. In this section, NBT stress is first decomposed into high gate voltage stress and high-temperature stress, and their effects on FDSOI PMOS in conjunction with irradiation are investigated separately. Finally, the simultaneous effect of high temperature, high gate voltage, and irradiation is investigated and the degradation of the devices under these cases is analyzed comparatively.

**Figure 7:**
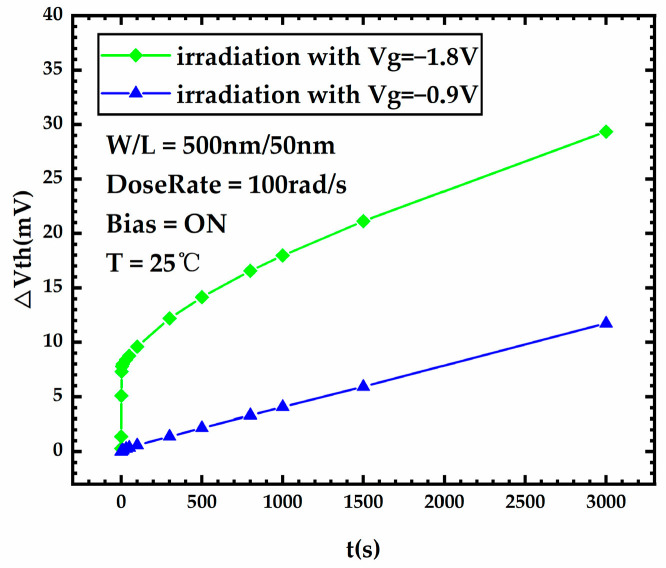
Threshold voltage degradation during stress for FDSOI PMOS devices under different gate voltages.

**Figure 8:**
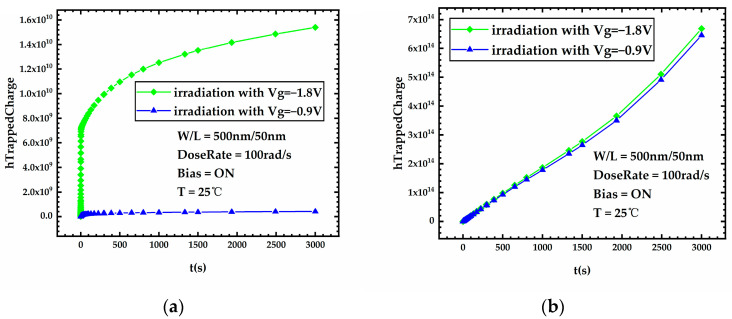
Variation of (**a**) front-gate interface trap charge and (**b**) oxide trap charge in the BOX layer versus stress time for FDSOI PMOS devices under different gate voltages.

Figure 7 presents the threshold voltage degradation during stress for FDSOI PMOS devices under different gate voltages. It can be seen that the devices under high gate voltage exhibit more severe threshold voltage degradation during irradiation compared to devices under low gate voltage. Figure 8a,b shows the variation curves of the front-gate interface trap charge and the oxide trap charge in the BOX layer versus stress time for FDSOI PMOS devices under different gate voltages, respectively. Figure 8 shows that after 300 Krad irradiation, the device under Vg = −1.8 V has a significant increase in the front-gate interface trap charge compared to the device under Vg = −0.9 V. Meanwhile, the oxide trap charge in the BOX layer of the device under Vg = −1.8 V increases insignificantly compared to the device under Vg = −0.9 V. The above analysis leads to the conclusion that high gate voltage has little impact on the oxide trap charge in the BOX layer generated during irradiation, and the front-gate interface trap charge induced by the high gate voltage contributes to severe threshold voltage degradation.

**Figure 9:**
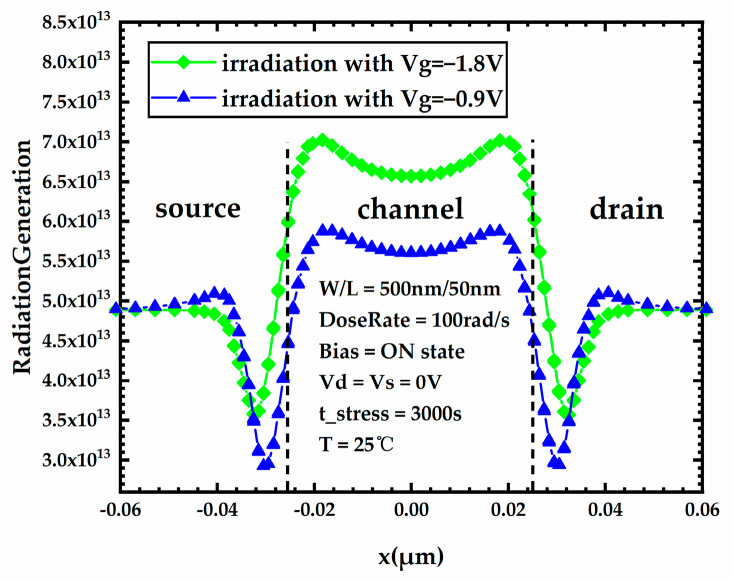
Lateral distribution of carrier generation rate in the BOX layer of FDSOI PMOS devices under different gate voltages during irradiation.

Figure 9 shows the lateral distribution of the carrier generation rate in the BOX layer of FDSOI PMOS devices under different gate voltages during irradiation. By analyzing Figure 8b and Figure 9, it can be noticed that high gate voltage results in an increased carrier generation rate in the BOX layer during irradiation; however, the increase in the number of oxide trap charges in the BOX layer generated during irradiation is very slight. This phenomenon can be explained by Equation (8). In Equation (8), $p_{1}$, $p_{2}$ are positive values and $p_{0}$ is a negative value. Thus, the higher the electric field strength, the smaller the trap’s capture cross-section for holes, which means a lower trap capture rate for holes. On the one hand, high gate voltage results in an increased carrier generation rate in the BOX layer during irradiation, which increases the number of oxide trap charges in the BOX layer, but on the other hand, high gate voltage also results in a decreased capture rate of traps for holes, which decreases the number of oxide trap charges in the BOX layer. These two factors compete with each other, which leads to a very slight change in the number of oxide trap charges in the BOX layer.

**Figure 10:**
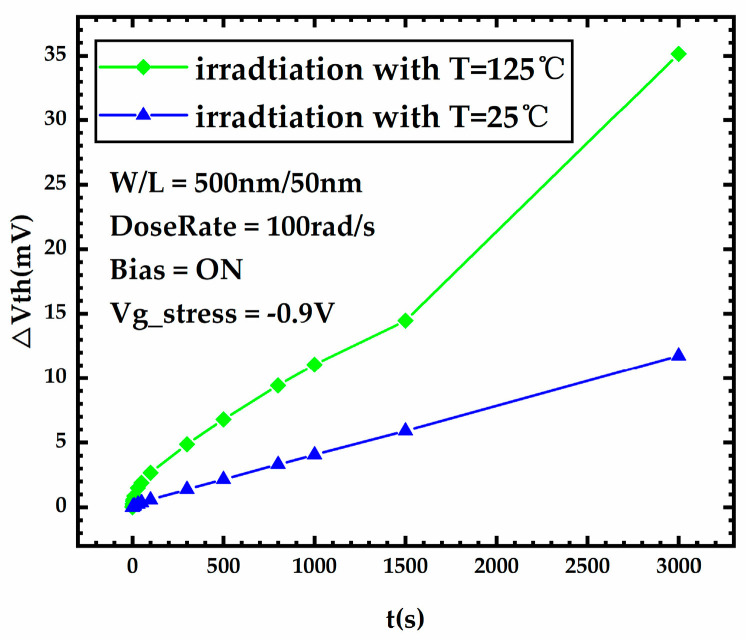
Threshold voltage degradation during stress for FDSOI PMOS devices under different temperatures.

Figure 10 shows the threshold voltage degradation during stress for FDSOI PMOS devices under different temperatures. It shows that the threshold voltage shift is higher for the device under 125 °C compared to devices under a low temperature. To understand this phenomenon, it is necessary to observe the trap charge of the device during irradiation.

**Figure 11:**
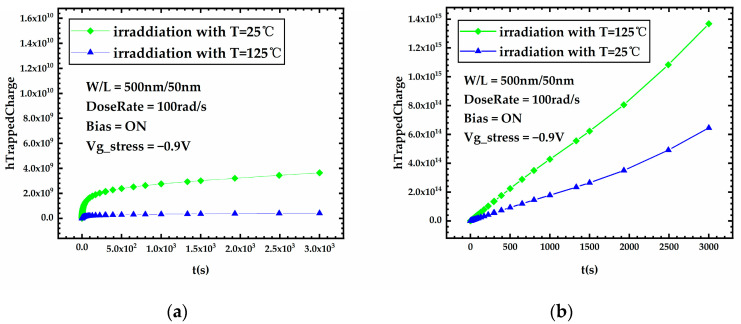
Variation of (**a**) the front-gate interface trap charge and (**b**) the oxide trap charge in the BOX layer versus stress time for FDSOI PMOS devices under different temperatures.

Figure 11a,b, shows the variation curves of the front-gate interface trap charge and the oxide trap charge in the BOX layer versus stress time for FDSOI PMOS devices under different temperatures, respectively. The high temperature causes a significant increase in the oxide trap charge in the BOX layer generated during irradiation, which is due to the fact that high temperature leads to a high capture rate of traps to holes. Equation (9) is the capture rate ($c_{V}^{p}$) of traps to holes in the oxide layer [21], which clearly demonstrates the positive correlation between the capture rate and the temperature.

(9) $$c_{V}^{p}=\sigma_{p}[\left(1-g_{p}^{J}\right)v_{th}^{p}+g_{p}^{J}J_{p}/q]$$

where $g_{p}^{J}$ is a constant between 0 and 1, $J_{p}$ is the hole current density, and $v_{th}^{p}$ is the thermal velocity, which in turn can be expanded as $v_{th}^{p}$ = $v_{0}^{p}$ $\sqrt{T/300K}$. As for the front-gate interface trap charge, it can be derived from Figure 11a that the front-gate interface trap charges generated under high-temperature irradiation also increased compared to those generated under low-temperature irradiation, which partly contributes to the higher threshold voltage shift of the device irradiated under high temperatures.

**Figure 12:**
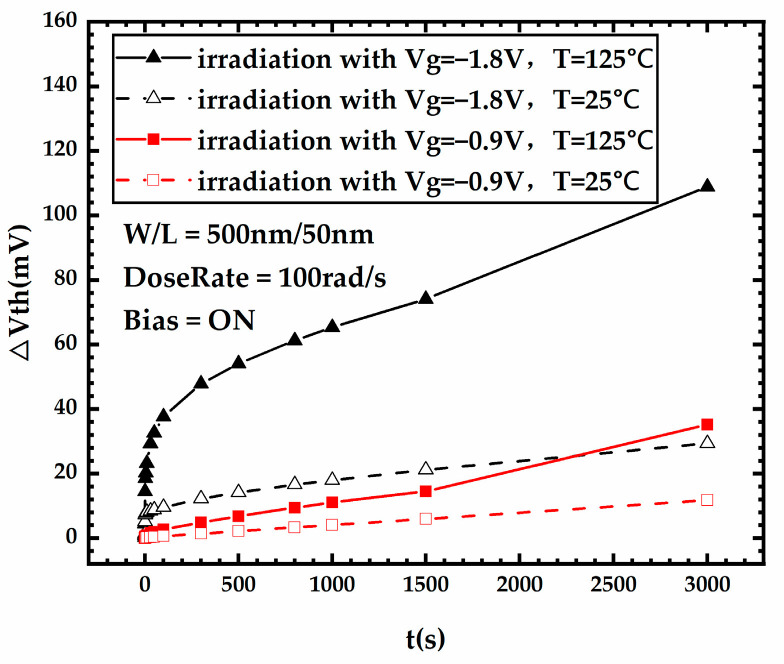
Threshold voltage degradation during stress for FDSOI PMOS devices under different temperatures and different gate voltages.

**Figure 13:**
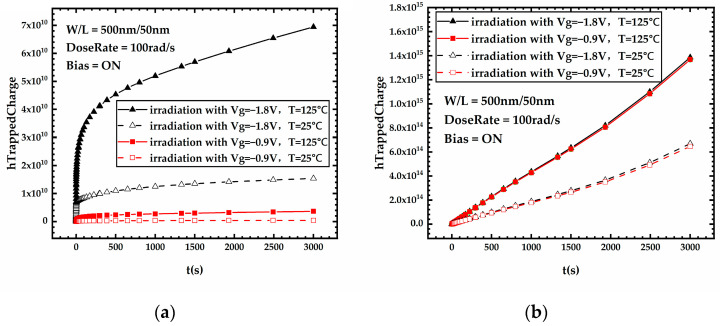
Variation of (**a**) the front-gate interface trap charge and (**b**) the oxide trap charge in the BOX layer versus stress time for FDSOI PMOS devices under different stress conditions.

Figure 12 shows the threshold voltage degradation during stress for FDSOI PMOS devices under different temperatures and different gate voltages, from which it can be observed that among four combinations of different gate voltages and temperatures, the threshold voltage degradation of the device irradiated under a high temperature and high gate voltage is the most severe one. Figure 13a,b shows the variation curves of the front-gate interface trap charge and the oxide trap charge in the BOX layer versus stress time for FDSOI PMOS devices under different stress conditions, respectively. According to Figure 13, under the combined effect of high temperature and high gate voltage, the impact of NBT stress is fully revealed; compared to other stress conditions, the generation of the front-gate interface trap charge increases extremely dramatically, and the oxide trap charge in the BOX layer also increases significantly. The threshold voltage shifts of the FDSOI PMOS irradiated under different stress conditions are displayed in Table 5. The high gate voltage increases the trap charge at the front-gate interface, the high temperature increases both the front-gate interface trap charge and the oxide trap charge in the BOX layer, and the irradiation under high temperature and high gate voltage achieved the effect of “one plus one over two”, which makes the degradation of the threshold voltage far more severe.

## 4. Conclusions

In this paper, the threshold voltage degradation of FDSOI PMOS under the coupling effect of NBTI and TID is investigated by simulation. The results of the situation of irradiation after NBT stress show that after NBT stress, the threshold voltage degradation during irradiation is weakened, which is caused by the recovery of the NBTI effect.

Then, the situation of the NBT stress and irradiation applied simultaneously to the FDSOI PMOS was investigated. According to the simulation result, both high gate voltage and high temperature make the threshold voltage degradation of the device during irradiation more severe. The high gate voltage results in a significant increase in the front-gate interface trap charge generated during irradiation, while the more severe degradation of the device under high-temperature irradiation is mainly attributed to the significantly increased oxide trap charge in the BOX layer. Under the simultaneous influence of high temperature and high gate voltage, both the front-gate interface trap charge and the oxide trap charge in the BOX layer generated during irradiation increase dramatically, which makes the threshold voltage degradation of the FDSOI PMOS far more severe.

## Figures and Tables

**Table 1:** Doping information of the FDSOI PMOS device model.

source drain gaussian doping peak value (cm^−3^)	1 × 10^20^
source drain gaussian doping minimum value (cm^−3^)	4 × 10^17^
LDD gaussian doping peak value (cm^−3^)	5 × 10^17^
LDD gaussian doping minimum value (cm^−3^)	1 × 10^16^
channel doping (cm^−3^)	1 × 10^15^
P type backplane doping (cm^−3^)	1 × 10^18^
work function (eV)	4.71

**Table 2:** Calibrated parameters of TDM.

Parameters	Value
$D_{0}$ (cm^2^/s)	5 × 10^−13^
$\varepsilon_{H}$ (eV)	0.37
$N_{H}^{0}$ (cm^−3^)	1 × 10^−15^
$v_{0}$ (s^−1^)	5 × 10^−12^
$N_{hb}^{0}$ (cm^−3^)	2 × 10^7^
N (cm^−3^)	5 × 10^13^
ρ	0.33
$\partial_{⊥}$ (eV∙cm^*ρ*^/V^*ρ*^)	−3.8 × 10^−3^
$\beta_{⊥}$ (cm/V)	−5 × 10^−8^

**Table 3:** Calibrated parameters of GRM.

Parameters	Value
$N_{ot}$ (cm^−3^)	8 × 10^17^
$g_{0}$ (rad^−1^∙cm^−3^)	1 × 10^13^
$E_{0}$ (V/cm)	0.1
$E_{1}$ (V/cm)	1.35 × 10^6^
m	0.9
$\sigma_{n}^{0}$ (cm^2^)	5 × 10^−14^
$\sigma_{p}^{0}$ (cm^2^)	5.1 × 10^−14^
$a_{1}$ (cm/V)	1.9 × 10^−4^
$a_{2}$ (cm/V)	1.2 × 10^15^
$p_{0}$	−1
$p_{1}$	0.55
$p_{2}$	1

**Table 4:** Bias conditions for PMOS during irradiation.

Bias State	V_G_	V_D_	V_S_	V_B_
OFF	0 V	−0.9 V	0 V	0 V
ON	−0.9 V	0 V	0 V	0 V
TG	0 V	−0.9 V	−0.9 V	0 V

**Table 5:** The threshold voltage shift of the FDSOI PMOS irradiated under different stress conditions.

Stress Condition	∆Vth
Irradiation with Dose = 300 Krad, Vg = −0.9 V, T = 25 °C	11.47 mV
Irradiation with Dose=300 Krad, Vg = −1.8 V, T = 25 °C	29.46 mV
Irradiation with Dose = 300 Krad, Vg = −0.9 V, T = 125 °C	35.16 mV
Irradiation with Dose = 300 Krad, Vg = −1.8 V, T = 125 °C	109.23 mV

## Data Availability

The original contributions presented in the study are included in the article, further inquiries can be directed to the corresponding author.
